# MetaPIGA v2.0: maximum likelihood large phylogeny estimation using the metapopulation genetic algorithm and other stochastic heuristics

**DOI:** 10.1186/1471-2105-11-379

**Published:** 2010-07-15

**Authors:** Raphaël Helaers, Michel C Milinkovitch

**Affiliations:** 1 Department of Biology, Facultés Universitaires Notre-Dame de la Paix (FUNDP), rue de Bruxelles 61, 5000 Namur, Belgium; 2 Laboratory of Artificial & Natural Evolution (LANE), Dept. of Genetics & Evolution Sciences III, 30, Quai Ernest-Ansermet, 1211 Genève 4, Switzerland

## Abstract

**Background:**

The development, in the last decade, of stochastic heuristics implemented in robust application softwares has made large phylogeny inference a key step in most comparative studies involving molecular sequences. Still, the choice of a phylogeny inference software is often dictated by a combination of parameters not related to the raw performance of the implemented algorithm(s) but rather by practical issues such as ergonomics and/or the availability of specific functionalities.

**Results:**

Here, we present MetaPIGA v2.0, a robust implementation of several stochastic heuristics for large phylogeny inference (under maximum likelihood), including a Simulated Annealing algorithm, a classical Genetic Algorithm, and the Metapopulation Genetic Algorithm (metaGA) together with complex substitution models, discrete Gamma rate heterogeneity, and the possibility to partition data. MetaPIGA v2.0 also implements the Likelihood Ratio Test, the Akaike Information Criterion, and the Bayesian Information Criterion for automated selection of substitution models that best fit the data. Heuristics and substitution models are highly customizable through manual batch files and command line processing. However, MetaPIGA v2.0 also offers an extensive graphical user interface for parameters setting, generating and running batch files, following run progress, and manipulating result trees. MetaPIGA v2.0 uses standard formats for data sets and trees, is platform independent, runs in 32 and 64-bits systems, and takes advantage of multiprocessor and multicore computers.

**Conclusions:**

The metaGA resolves the major problem inherent to classical Genetic Algorithms by maintaining high inter-population variation even under strong intra-population selection. Implementation of the metaGA together with additional stochastic heuristics into a single software will allow rigorous optimization of each heuristic as well as a meaningful comparison of performances among these algorithms. MetaPIGA v2.0 gives access both to high customization for the phylogeneticist, as well as to an ergonomic interface and functionalities assisting the non-specialist for sound inference of large phylogenetic trees using nucleotide sequences. MetaPIGA v2.0 and its extensive user-manual are freely available to academics at http://www.metapiga.org.

## Background

Phylogeny inference allows, among others, detecting orthology/paralogy relationships among gene family members (*e.g*., [1,2]), estimating divergence times and evolutionary rates (*e.g*., [3-5]), reconstructing ancestral sequences (*e.g*., [6-9]), identifying molecular characters constrained by purifying selection or prone to positive selection (*e.g*., [10]), uncovering hidden biodiversity (*e.g*., [11]), and mapping the evolution of morphological, physiological, epidemiological, biogeographical and even behavioral characters [12,13]. Molecular phylogeny inference is now a mature science, and an important part of the maturation process pertained to the realization (in the last 10 years) that the quest for the Holy Grail of absolute best tree should be abandoned for a much more meaningful goal: the inference of clades and trees robustness. Still, that objective remained intractable in practice because of *(a) *the NP-hard nature of optimality-criterion-based phylogeny inference (*i.e*., no algorithm can solve it in polynomial time; [14,15]) and *(b) *the computing-time requirements of using complex substitution models (and rate heterogeneity across sites) in the framework of what had been identified as the probable most robust optimality criterion: Maximum Likelihood (ML; [16-18]). Today large phylogeny inference is incorporated, across biological disciplines, as an essential step in most comparative studies involving nucleotide or protein sequences. This has been made possible thanks to both theoretical and practical developments.

First, one key advance that made large phylogeny inference tractable is the implementation in this field of stochastic heuristics with inter-step optimization, *i.e*., a family of approaches that existed for decades in physics and computer science and explore multidimensional solution spaces in a much more efficient manner than the older intra-step optimization hill-climbing methods. Indeed, in the latter, one prime parameter (typically, the topology of the tree) is modified and all other parameters are optimized before the new solution is evaluated whereas, in stochastic heuristics, all free parameters are optimized while the search proceeds. Inter-step optimization methods include MCMC approximations of the Bayesian approach [19,20], stochastic simulated annealing [21], and genetic algorithms [22-26]. The efficiency of stochastic heuristics is quite counter-intuitive but can be explained by several factors: *(a) *poorer solutions are accepted with a non-null probability (contrary to hill-climbing that strictly restricts moves toward better likelihood values) such that valleys in likelihood space can eventually be crossed; *(b)*, parameters are not over-optimized (*e.g*., starting and intermediate trees are generally largely sub-optimal, hence, optimizing model parameters on these topologies is a clear example of over-fitting). We think that avoiding over-optimization at every topology evaluation generates a flatter likelihood space shape, such that valleys are more easily crossed and local optima more easily escaped. This suggestion however requires further investigation.

Second, several stochastic methods have been incorporated into robust application softwares. The importance of that point should not be underestimated. For example, the recent success of Bayesian methods is probably due as much to its incorporation into a robust and efficient software (MrBayes; [27]) as to the theoretical appeal of generating marginal posterior probabilities [19]. The software RaxML [28], enjoys well-deserved popularity because it is one of the fastest ML phylogeny inference programs available to date (despite that it does not incorporate stochastic methods) thanks to the implementation of approximations to rate heterogeneity across sites and of smart computer science tricks speeding up likelihood computation: optimized parallel code and 'Subtree Equality Vectors' (*i.e*., the extension of character compression to the subtree level). Similarly, highly efficient parallel code has recently been implemented for the evaluation of phylogenies on graphics processing units (GPUs), resulting in about 100-fold speed increase over an optimized CPU-based computation [29]. This efficient use of new hardware, existing stochastic heuristics (in this case, an MCMC approach in a Bayesian framework), and smart code parallelization for efficient harnessing of the hundreds of GPU processing cores, allowed the authors to successfully use a 60-state codon model on a dataset of 62 complete mitochondrial genomes [29].

The availability of multiple excellent softwares implementing different robust heuristics is clearly an asset for the end user: reliable results might be identified because they remain stable across softwares and methods. However, most users chose one single main software for their analyses, and this choice is sometimes dictated by availability of functionalities of importance but that do not pertain to the performances of the specific heuristic implemented (*e.g*., ability to perform batch analyses, availability of GTR nucleotide substitution model [30] or of rate heterogeneity [31-33], possibility to partition data). Finally, given that the need to infer large trees is critical in multiple biological disciplines, the non-specialist can be baffled by the large number of available heuristics, parameters, and softwares, such that the most user-friendly tools are often preferred even if more robust or more efficient (but less user-friendly) softwares are available. There is therefore a challenge to supply softwares that are easy to use for the non-specialist, provide flexibility for the specialist, and allow fast and robust inference for both.

The Metapopulation Genetic Algorithm (MetaGA; [23]) is an evolutionary computation heuristic in which several populations of trees exchange topological information which is used to guide the GA operators for much faster convergence. Despite the fact that the metaGA had been implemented in a simple and unoptimized software (metaPIGA v1) together with simple nucleotide substitution models, an approximate rate heterogeneity method, and only a low number of functionalities, is has been shown as one of the most efficient heuristics under the ML criterion [23,34,35]. Furthermore, it has been suggested that multiple metaGA searches provide an estimate of the posterior probability distribution of possible trees [23] although this proposition clearly warrants much further investigation. Here, we present MetaPIGA-2.0 the first phase of a robust implementation of the MetaGA (and other stochastic methods such as a classical Genetic Algorithm and Simulated Annealing) together with complex substitution models, rate heterogeneity, and high parameterization for the phylogeneticist, as well as an ergonomic interface and easy-to-use functionalities for the non-specialist.

## Implementation and Results

### ML framework

Trees are estimated in MetaPIGA-2.0 with the Maximum Likelihood criterion (ML) using any of 5 nucleotide substitution models ([2] and refs therein, [30]): Jukes Cantor (JC), Kimura's 2 parameters (K2P), Hasegawa-Kishino-Yano 1985 (HKY85), Tamura-Nei 1993 (TN93), and General Time Reversible (GTR). Analyses can be performed with rate heterogeneity among sites using a proportion of invariant sites (*Pinv*) [33] and/or a discrete Gamma distribution of rates (*γ*-*distr*) [31,32]. All parameters of the model (transition/transversion ratio or components of the rate matrix, the shape parameter of the *γ*-*distr*, and *Pinv*) can be set by the user or estimated from a Neighbor Joining (NJ) tree [36]. The same parameters plus branch lengths and among-partition relative rates can experience intra-step optimization either periodically during the search and/or at the end of the search.

Datasets can be partitioned into character sets ("charsets") either using a graphical tool (see below) or by writing the corresponding commands in a batch file. In MetaPIGA-2.0, we assume that all partitions evolve on the same topology (we therefore consider, like in the vast majority of phylogeny inference programs, that the analysis is performed on a non-recombining piece of DNA, such that the phenomena of hybridization and incomplete lineage sorting are ignored), but all other parameters (base frequencies, substitution matrix rates, shape parameter of *γ*-*distr*, and *Pinv*) are optimized separately for each partition. Among-partition rate variation parameters are introduced in the likelihood equation as a factor that modifies branch length for the corresponding partition. Branch lengths are optimized as usual, but the relative rates of partitions are optimized separately (with the constraint that the weighted average of among-partitions rates is 1; weighting is according to each partition size).

### Tools shared among heuristics

Phylogeny estimation is an NP-hard problem [14,15], with unknown search space topography. MetaPIGA-2.0 implements four different heuristics for searching solution space (see below). A set of tools is shared by all these heuristics: the starting tree generators, the operators, and some of the stopping rules.

A Tree Generator is used to produce the starting tree(s) either as NJ tree(s) [36] or as random tree(s) (*i.e*., with random topology and random branch lengths) or as "Loose Neighbor Joining" (LNJ) tree(s), *i.e*., a pseudo-random topology (modified from [23]). For generating a LNJ tree, the user specifies a proportion value (*p *= [0-1]) and, at each step of the NJ algorithm, the two nodes to cluster, instead of corresponding to the smallest distance value, are randomly chosen from a list containing the $\frac{NTax(Ntax-1)p}{2}$ smaller distances, where *NTax *is the number of taxa (sequences) in the dataset. Branch lengths are computed as in the NJ method [36]. In other words, the LNJ tree is a NJ tree with some topology randomization which amount is defined by the user. This approach is a particularly useful compromise between random starting topologies (*p *= 1) that require long runs of the heuristic for optimization, and a good but fixed topology (the NJ tree, *i.e*., *p *= 0) that is prone to generate solutions around a local optimum. The distance matrix used for building NJ or LNJ starting trees can be computed using any of the 5 substitution models (see above) and with or without *Pinv *and/or *γ*-*distr*. Arbitrary starting trees can also be imported by the user.

At the core of all stochastic heuristics are the Operators, *i.e*., the topology and parameters' modifiers allowing the heuristic to explore solution space. In MetaPIGA-2.0, we implemented 5 operators for perturbing tree topology (Nearest Neighbor Interchange, *NNI*; Subtree Pruning Regrafting, *SPR*; Tree Bisection Reconnection, *TBR*; Taxa Swap, *TXS*; Subtree Swap, *STS*; see [37] and [23] for details) and 6 operators for perturbing model parameters (branch lengths, internal branch lengths, rate matrix parameters, *γ*-*distr *shape parameter, *Pinv*, and among-partitions rate variation). These operators can be used in any combination, either at equal or user-defined frequencies. The user can choose for these frequencies to change dynamically during the search, *i.e*., MetaPIGA periodically evaluates the relative gains in likelihood produced by each operator and adjusts their frequencies proportionally. Minimum frequencies can be set such that operators that are inefficient early in the search remain available for increased use later in the search.

All stochastic heuristics require a stopping condition. In MetaPIGA-2.0, the user can choose any combination of the following criteria: number of steps, elapsed time, likelihood stability, and convergence of branch support distribution (for replicated searches). When using the metaGA heuristic, one can additionally use a stopping condition, within each replicate, based on convergence of the populations of solutions (see below).

### The heuristics

We implemented four heuristics in MetaPIGA-2.0: a Simulated Annealing algorithm (SA; [21]), a classical Genetic Algorithm (GA; [22,24,25,38]) and the metapopulation Genetic Algorithm based on the Consensus Pruning principle (metaGA; [23]). As a reference, we also implemented a simple Hill Climbing (HC) algorithm that generates a new solution tree at each step (using available operators) and accepts it only if its likelihood is better than the current solution. HC algorithms are fast but tend to generate solutions trapped in local optima and are therefore highly dependent on the starting tree localization in tree space as well as on the (unknown) tree space topography.

#### The simulated annealing (SA)

The SA algorithm uses statistical mechanics principles to solve combinatorial optimization problems [39]; *i.e*., it mimics the process of minimal energy annealing in solids. SA starts with some initial state (the starting tree) and randomly perturbs that solution (using available tree operators). If the new state is better (lower energy, better likelihood), it is kept as the new current state; if the new state is worse (higher energy, worse likelihood), it is accepted as the current state with the Boltzmann Probability *e*^Δ*E/T*^, where Δ*E* is the negative difference in energy (here, the difference of likelihood) between the two states, and *T *is the so-called 'temperature' of the system. If *T *is lowered slowly enough, the algorithm is guaranteed to find the optimal solution. The obvious asset of the algorithm is its ability to momentarily accept suboptimal solutions, allowing it to escape local optima whereas its obvious drawback is the difficulty to define the shape and speed of the "cooling schedule" (*i.e*., the rate of the decrease in *T*). Efficient schedules highly depend on the dataset. We implemented 14 highly-parametrized cooling schedules in MetaPIGA-2.0, including one specifically developed for phylogeny inference [40]. The user can control all cooling schedule parameters: the starting temperature computation method, the maximum acceptance probability, the temperature decrease frequency, and the possibility of 'reheating'.

#### The Genetic Algorithm (GA)

The GA is an evolutionary computation approach that implements a set of operators mimicking processes of biological evolution such as mutation, recombination, selection, and reproduction (*e.g*., [41]). After an initial step of generating a population of trees, the individuals (specific trees and model parameters) within that population are *(i) *subjected to mutation (a stochastic alteration of topology, of branch lengths or of any model parameter) and/or recombination, and *(ii) *allowed to reproduce with a probability that is a function of their relative fitness value (here, their likelihood). Because selection preferentially retains changes that improve the likelihood, the mean score of the population improves across generations. However, because sub-optimal solutions can survive in the population (with probabilities that depend on the selection scheme), the GA allows, in principle, escaping local optima. In MetaPIGA-2.0, we implemented 5 alternative selection schemes ("*Rank*", "*Tournament*", "*Replacement*", "*Improve*", and "*Keep the Best*", see [23]) and one recombination scheme where each sub-optimal individual has a probability (determined by the user) to recombine with a better individual. Recombination is performed by exchanging subtrees defined by one (if any) of the identical taxa partitions in the two parental trees (*i.e*., one internal branch that defines subtrees including the same taxa but with potentially different sub-topologies). A recombination can be viewed as a large number of simultaneous topological mutations. Beside the selection scheme, the major parameter in the GA is the population size (set by the user).

#### The metapopulation Genetic Algorithm (metaGA)

This approach relies on the coexistence of *P *interacting populations [23] of *I *individuals each (*P *and *I *defined by the user): the populations are not fully independent as they cooperate in the search for optimal solutions. Within each population, a classical GA is performed: trees are subjected to evaluation, selection (5 alternative selection schemes are available as in the GA, see above), and mutation events. However, all topological operators are guided through inter-population comparisons defined and controlled by '*Consensus Pruning' *(CP; [23]): topological consensus among trees across populations defines the probability with which different portions of each tree are subjected to topological mutations. These comparisons allow the dynamic differentiation between internal branches that are likely correct (hence, that should be changed with nil or low probability) and those that are likely incorrect (hence, that should be modified with high probability). Although CP allows for the elaboration of many alternative inter-population communication procedures [23], we implemented in MetaPIGA-2.0 the two that we identified (data not shown) as the most useful: '*Strict CP' *(internal branches shared by all trees across all populations cannot be affected by topological mutations; all other internal branches are unconstrained) and '*Stochastic CP' *(topological mutations affecting a given branch are rejected with a probability proportional to the percentage of trees across all populations that agree on that branch).

As constraining entirely an internal branch from being affected by topological mutations necessarily increases the likelihood to be trapped in a local optimum, a tolerance parameter *t *(defined by the user) is implemented, allowing any internal branch to be affected with a probability *t *even if the branch is shared by all trees. The user of MetaPIGA-2.0 has the choice between a '*blind' *and a '*supervised' *procedure for handling constrained partitions. In the former, a topological mutation that affects a constrained branch is simply aborted and the tree is left unchanged, whereas in the latter, topological operators exclusively target branches in a pool of acceptable (unconstrained) candidates.

**Figure 1:**
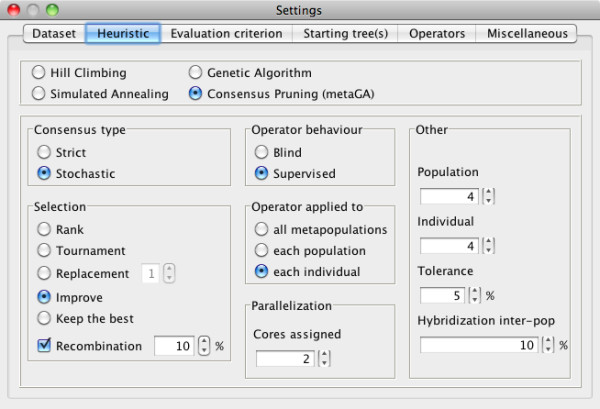
**The MetaPIGA-2.0 heuristic setting window**. The user can set the parameters of the chosen heuristic: here, for the metaGA, the consensus type, the selection scheme, the operator behavior, the number of cores/processors (here, 2 cores) onto which the populations are distributed, the number of populations and the number of individuals per population, the tolerance parameter (frequency with which internal branches are affected by mutational operators even if that branch is present in all trees across all populations), and the frequency of hybridization among populations. See text for details.

The MetaGA allows for two, non-mutually exclusive, recombination flavors: '*intra-population recombination' *where each sub-optimal individual at each generation has a probability (instead of being mutated) to recombine with a better individual from that population (as in the GA above), and '*inter-population hybridization' *(Figure 1) where, at each generation, there is a probability (defined by the user) that all sub-optimal individuals from one random population are recombined with one individual from another population; sub-optimal individuals from other populations experience the normal mutation procedure.

Comparing, across generations, the frequencies of internal branches shared among the P*I trees (irrespective of how the trees are assigned to populations) provides a means for assessing whether the populations converge towards a stable set of solutions, *i.e*., towards a consensus with stable branch frequencies. Hence, a stopping rule, not available to other heuristics, can be used under CP: the user can choose to stop the search when a series of mean relative error (MRE) values remains, across generations, below a threshold defined by the user. To increase independence among samples, consensus trees are sampled every *n *> 1 (*i.e*., non-successive) generations. For example, given two consensus tree, *T*_*i *_and *T*_*j*_, corresponding to the consensuses among the P*I trees at generations 5000 and 5005, respectively, the MRE is computed as follows:

$MRE(T_{i},T_{j})=\frac{\sum_{p=1}^{nPartition}\left|\frac{\Phi_{T_{i}}^{p}-\Phi_{T_{j}}^{p}}{\max(\Phi_{T_{i}}^{p},\Phi_{T_{j}}^{p})}\right|}{nPartition}$, where *nPartition *is the sum of taxa bi-partitions observed in *T*_*i *_and *T*_*j *_(but identical partitions are counted once), and $\Phi_{Ti}^{p}$ and $\Phi_{Tj}^{p}$ are the consensus values of bi-partition *p *in trees *T*_*i *_and *T*_*j*_, respectively. Note that $\left|\frac{\Phi_{T_{i}}^{p}-\Phi_{T_{j}}^{p}}{\max(\Phi_{T_{i}}^{p},\Phi_{T_{j}}^{p})}\right|=1$ if either both $\Phi_{Ti}^{p}$ and $\Phi_{Tj}^{p}$ are nil, or if the corresponding internal branch does not exist in either *T*_*i *_or *T*_*j*_. Internal branches that are absent from both *T*_*i *_and *T*_*j *_are not considered. If the MRE(gen5000, gen5005) is above the user-defined threshold (*e.g*., 5%), it is discarded and a new MRE is computed for the comparison of generations 5005 and 5010. On the other hand, if MRE(gen5000, gen5005) is below the threshold, a counter is incremented and a new MRE is computed for the comparison of generations 5000 with the next sample (here, corresponding to generation 5010). The user defines for how many samples the MRE must remain below the specified threshold before the search stops.

### Replicates

For any heuristic chosen by the user in MetaPIGA-2.0, the search can be repeated many times, generating a majority-rule consensus tree among the replicates. Note that when the metaGA is the selected heuristic, it has been suggested that the frequencies of clades in the among-replicates consensus might approximate the corresponding posterior probabilities [23]. The user can either fix the number of replicates, or specify a range of minimum and maximum number of replicates and let MetaPIGA-2.0 stop automatically, exploiting the MRE metric in a similar way as the consensus across populations in a single metaGA search (see above). Here, however, the MRE is computed using consensuses across replicates, *i.e*., *T*_*i *_is the consensus among the final trees that have been obtained in replicates 1 to *i*. No additional replicate is produced when the MRE among *N *replicates (one can use consecutive replicates because they are independent) remains below a given threshold. As an example, if *N *is set to 10, and the first MRE below the user-defined threshold (*e.g*., 5%) involves replicates 1-241 and 1-242, the MRE is computed 9 additional times, *i.e*., between the reference consensus *T*_*1-241 *_and *T*_*j*_, for *j *corresponding to replicates 1-243, then 1-244, then 1-245, etc. The search stops if the inter-replicates MRE remains below 5% for 10 consecutive replicates. On the other hand, the counter is reset to zero as soon as the MRE exceeds 5%, and the new reference tree for computing MRE is then set to *T*_*1-current replicate*_.

The inter-generations (= intra-replicate) MRE stopping rule can be used in combination with the inter-replicate MRE stopping rule, letting MetaPIGA decide both when to stop each replicate and when to stop executing additional replicates (*i.e*., when to stop the entire analysis).

### Language, formats, and interface

MetaPIGA-2.0 is written in Java 1.6 such that the single code runs on 32 and 64-bits platforms under MacOS X, Linux, and Windows. Computing and storing the likelihood of large trees requires large amount of Random-Access Memory (RAM). Whereas 32-bits systems can allocate a maximum of ~2Gb of memory to the Java Virtual Machine (JVM), 64-bits systems are virtually limited only by the amount of memory installed on the computer (as the theoretical limit is about 18 billions gigabytes). MetaPIGA-2.0 uses the Java Multi-Threading technology to take advantage of multiprocessor and multicore computers, such that some tasks can be run in parallel. As replicates are independent, they are particularly prone to parallelization: any number of different cores/processors can be assigned to different replicates. In addition, the metaGA heuristic itself is well suited to parallel implementation because processes such as mutation, selection, and likelihood computation are unrelated to CP and are therefore independent across populations. Hence, different metaGA populations can be distributed to different cores/processors. Parallelization of metaGA populations can be combined with parallelization of replicates (*e.g*., 16 cores allow running simultaneously 4 metaGA replicates with 4 populations/replicate).

MetaPIGA-2.0 uses standard formats: reading and writing datasets in Nexus format [42] and trees in Newick format http://evolution.genetics.washington.edu/phylip/newicktree.html. All search settings can be saved in a metaPIGA block incorporated into the Nexus file, allowing easy management and command line runs. A Nexus file without a metaPIGA block will be correctly interpreted by MetaPIGA-2.0 and will run with default parameters.

**Figure 2:**
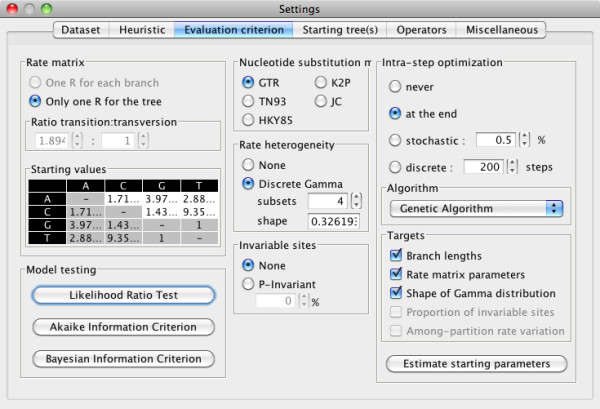
**The MetaPIGA-2.0 model setting window**. The user can choose a substitution model and set the corresponding parameters: here, the GTR model (and estimated starting values of the rate matrix) with rate heterogeneity (discrete Gamma model) and no proportion of invariable sites has been selected automatically after performing a likelihood ratio test (lower left buttons). The user can also choose how and when intra-step optimization of target parameters (here, branch lengths, rate matrix parameters, and the alpha shape parameter of the Gamma distribution) will be performed (here, at the end of the search, using a genetic algorithm). Note that, as the metaGA is a stochastic heuristic, most of the parameters optimization occurs inter-step, *i.e*., across generations under the effect of operators (see Figure 3).

**Figure 3:**
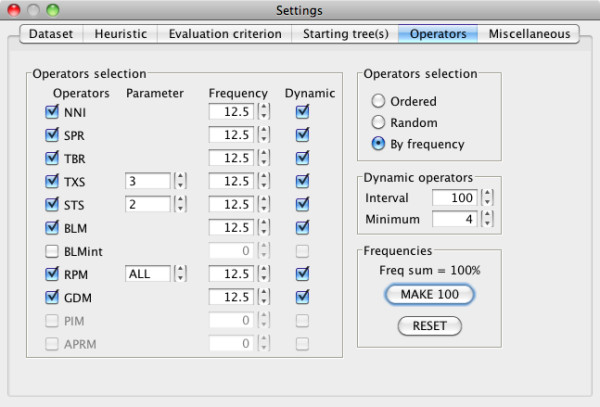
**The MetaPIGA-2.0 operators setting window**. The user selects the operators (affecting topology, branch lengths, and model parameters), their frequencies (unless they are ordered or randomly selected; upper right radio-button panel) and whether their frequencies are dynamically adapted (here, every 100 generations but never set below 4%) depending on their relative efficiencies in improving the best-tree likelihood. See text for details.

**Figure 4:**
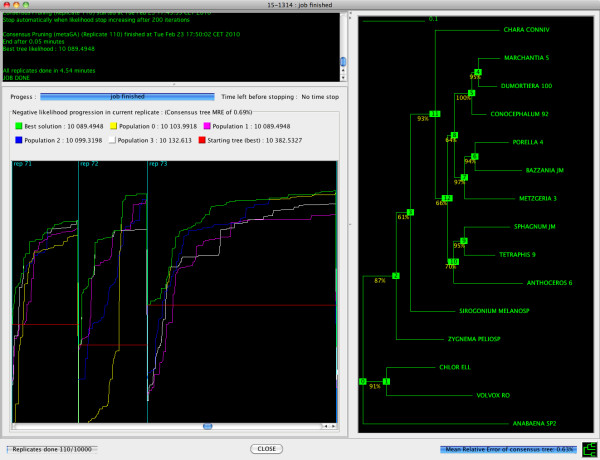
**The MetaPIGA-2.0 run window**. Here, a metaGA search with multiple replicates has been chosen. Hence, the run window shows, for 3 successive replicates (lower left panel), the current best-tree likelihood progression in each population, as well as (right panel) the current topology, metaGA branch support values, and average branch lengths of the consensus among the best trees (one for each population) from all replicates.

MetaPIGA-2.0 can be run in command line but it also offers an extensive graphical user interface (GUI) for access to all search settings: defining and managing charsets; including/excluding taxa, characters, and charsets; managing dataset partitions; choosing and parametrizing heuristics (Figure 1); defining substitution models and their parameters (Figure 2); choosing starting tree options; controlling operators (Figure 3); defining stop criteria and replicates. All settings are associated with an interactive "mouse-over" help system. MetaPIGA-2.0 also implements three statistical methods (Figure 2) for selecting the substitution model that best fits the data ([43]; and refs therein): the Likelihood Ratio Test, the Akaike Information Criterion, and the Bayesian Information Criterion. The MetaPIGA-2.0 GUI provides a detailed run window showing graphs specific to the chosen heuristic (*e.g*., for a metaGA search with replicates: current best likelihood progression of each population as well as the current topology, branch support values, and the average branch lengths of the consensus tree; Figure 4).

Batch files are particularly useful for running sequentially a single data set under multiple different settings and/or several datasets with the same settings. MetaPIGA-2.0 supports the use of batch files which can be written manually or generated using tools available in the GUI: datasets and their settings can be duplicated, settings can be copy-pasted from one dataset to another, and multiple combinations of datasets and settings can be saved in a batch file that can be run either in the GUI (with various graphical information on search progress) or using command line.

**Figure 5:**
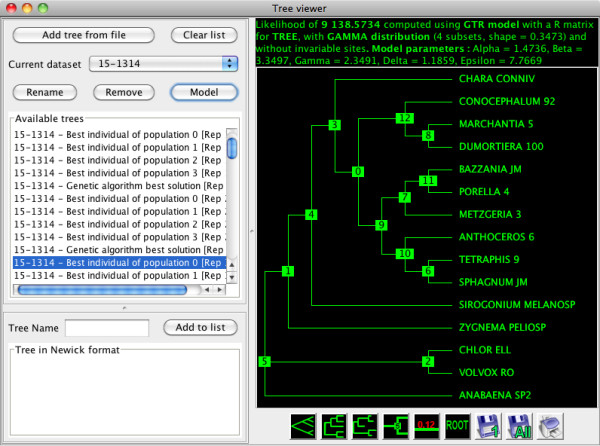
**The MetaPIGA-2.0 Tree Viewer**. The tree selected in the list of available trees (left panel) is show in the right panel with its likelihood and model parameters. Trees can be viewed, rerooted, and printed. Likelihood can be recomputed after changing the substitution model or optimizing model parameters. Various tools allow modifying the list of trees.

Input and result trees are manipulated in Newick format but visualized graphically in the GUI and can be exported for other programs. MetaPIGA-2.0 also integrates a Tree Viewer (Figure 5) that allows viewing, rerooting, and printing trees as well as computing the likelihood of any tree (under any substitution model) and optimizing its model parameters. Three other tools are implemented in MetaPIGA-2.0: a tree generator (using the starting tree settings), a consensus builder (using user trees and/or trees saved in the Tree Viewer), and a memory setting tool defining the maximum amount of memory allocated to the program.

## Discussion and Conclusions

The metaGA resolves the major problem inherent to classical GA approaches: should one use a soft or a stringent selection scheme? Indeed, strong selection produces good solutions in a short computing time but tends to generate sub-optimal solutions around local optima, whereas mild selection schemes considerably improve the probability to escape local optima and find better solutions but greatly increase computing time. As the metaGA involves several parallel searches, initial inter-population variation can be very high (especially if random or LNJ pseudo-random starting trees are used), and somewhat maintained during the search, even under extreme intra-population selection.

Although the metaGA has been shown to perform very well [23,34,35], it had not been implemented together with complex substitution models, discrete Gamma rate heterogeneity, and the possibility to partition data. Here, we performed such an implementation together with a hill climbing, a classical GA, and a SA algorithm. This implementation into a single software will allow a rigorous identification of the optimal parameters' values under each of these heuristics as well as a meaningful comparison of performances among these algorithms. Comparing the performances (speed and accuracy) between metaPIGA-2.0 and other popular softwares such as, among others, MrBayes [27], RaxML [28], Garli [38], and PhyML [44] is well beyond the scope of the present manuscript. However, our preliminary analyses (data not shown) with large datasets indicate that metaPIGA-2.0 and MrBayes-3.1.2 generate very similar candidate trees and consensus cladograms (under stopping rules based on the inter-replicate MRE metric or average standard deviation of split frequencies, respectively) and require similar running times.

An in-depth assessment of the statistical significance of metaGA branch support values is warranted. There might be some correspondence between metaGA branch support values and posterior probabilities [23] but theoretical and additional empirical analyses are required. For example, it would be important to asses how changing metaGA (versus MCMC) settings would affect sampling and estimates of probability distributions.

MetaPIGA-2.0 will constitute a platform on which we will incorporate additional functionalities (*e.g*., amino-acid and codon substitution models, and inference of ancestral sequences), improve performances (*e.g*., by parallelization on graphics processing units), identify optimal combinations of default parameters values, improve current heuristics, and possibly combine them for the development of higher-level metaheuristics. Meanwhile, MetaPIGA-2.0 already gives access both to high customization for the phylogeneticist, as well as to an ergonomic interface and functionalities assisting the non-specialist for sound inference of large phylogenetic trees using nucleotide sequences.

## Availability and Requirements

**Project name: **MetaPIGA-2.0

**Project home page: **http://www.metapiga.org and http://www.lanevol.org

**Operating systems: **Platform independent

**Programming language: **Java

**Other requirements: **Java 1.6 virtual machine

**License: **Free for Academics, license needed for non-academics.

## Authors' contributions

MCM conceived and designed the software, and wrote the manuscript. RH improved the design of many functionalities and wrote the code. Both authors read and approved the final manuscript.

## Authors' Information

MCM heads the Laboratory of Artificial & Natural Evolution (LANE) at the University of Geneva (Switzerland), and works on various aspects of Evo-Devo, phylogenomics, phyloinformatics, experimental evolution, and conservation genetics. RH is a computer scientist in the department of Biology of Namur University (Belgium) and specializes in the development of bioinformatics tools.
